# Synthesis, Characterization and Sensor Application of Novel PCL-Based Triblock Copolymers

**DOI:** 10.3390/polym17070873

**Published:** 2025-03-25

**Authors:** Murat Mısır

**Affiliations:** Faculty of Engineering and Architecture, Kırşehir Ahi Evran University, 40100 Kırşehir, Türkiye; murat.misir@ahievran.edu.tr

**Keywords:** ring-opening polymerization, reversible addition–fragmentation chain transfer polymerization, sensor ability

## Abstract

In this study, novel triblock copolymers, including poly(*N*-isopropylacrylamide)-*block*-poly(*ε*-caprolactone)-*block*-poly(*N*-isopropylacrylamide) (PNIPAM-*b*-PCL-*b*-PNIPAM), poly(*N*-vinyl-pyrrolidone)-block-poly(*ε*-caprolactone)-block-poly(*N*-vinyl-pyrrolidone) (PNVP-*b*-PCL-*b*-PNVP), poly(*N*-isopropylacrylamide-*co*-*N*,*N*-dimethylaminoethyl methacrylate)-*block*-poly(*ε*-caprolactone)-*block*-poly(*N*-isopropylacrylamide-*co*-*N*,*N*-dimethylaminoethyl methacrylate) (P(DMAEMA-*co*-NIPAM)-*b*-PCL-*b*-P(NIPAM-*co*-DMAEMA)), and poly(*N*,*N*-dimethylacrylamide)-*block*-poly(*ε*-caprolactone)-*block*-poly(*N*,*N*-dimethylacrylamide) (PDMA-*b*-PCL-*b*-PDMA), were synthesized via a combination of ring-opening polymerization (ROP) and reversible addition–fragmentation chain transfer (RAFT) polymerization. The synthesis was performed using novel bifunctional PCL-based RAFT macro chain transfer agents (macroCTAs; MXTPCL-X1 and MXTPCL-X2) with a *m*-xylene-bis(2-mercaptoethyloxy) core. Initially, *m*-xylene-bis(1-hydroxy-3-thia-propane) (MXTOH), which has not previously been used in lactone polymerization, was synthesized via the reaction of *α*,*α*′-dibromo-*m*-xylene with 2-mercaptoethanol in the presence of sodium in ethanol. Subsequently, Sn(Oct)_2_-catalyzed ROP of *ε*-caprolactone (*ε*-CL) using MXTOH as an initiator yielded PCL-diol (MXTPCLOH). The resulting PCL-diol underwent further functionalization through esterification and substitution reactions, leading to the formation of PCL-based RAFT macroCTAs. Triblock copolymers were synthesized using these macroCTAs with AIBN as an initiator. The synthesized products, along with their intermediates, were characterized using FTIR and ^1^H NMR spectroscopy. The number average molecular weight (M_n_) and polydispersity index (*Ð*) of PCL-based macroCTAs were determined by using GPC analysis. The sensor capabilities of the synthesized novel triblock copolymers were investigated on the determination of syringic acid and it was determined that the most sensitive polymer was PNVP-*b*-PCL-*b*-PNVP (MXTP2). The working range was between 1.5 µg/mL and 15 µg/mL and the limit of detection (LOD) was found to be 0.44 µg/mL using DPV on MXTP2 polymer sensor.

## 1. Introduction

Poly(*ε*-caprolactone) (PCL), an aliphatic polyester synthesized by ring-opening polymerization (ROP) of *ε*-CL, is a semi-crystalline, hydrophobic polymer with good mechanical properties for applications in industrial and biomedical fields [1,2,3,4]. Aliphatic biodegradable polyesters, such as poly(*ε*-caprolactone) (PCL), poly(lactic acid) (PLA), and their copolymers, are interesting polymers used in drug delivery systems [5], medical devices [6], biomedical and pharmaceutical materials [7]. Ring opening polymerization (ROP), the preferred technique for preparation of aliphatic polyesters, is a chain growth polymerization method that can proceed in a controlled or living manner, depending on the monomer and initiator/catalyst system [8]. Molecular weight and dispersity (*Ð*), which provide information about the chain length to determine the physical properties of the aliphatic polyesters, can be controlled with organometallic-catalyzed ROP of cyclic esters [9]. Tin(II) 2-ethylhexanoate (Sn(Oct_2_), is the most well-known and used catalyst in ROP of lactones and lactides, has low toxicity to humans, and has been accepted as a food stabilizer, like SnCl_2_, by the Food and Drug Administration (FDA) [10].

Block copolymers (BCPs) are a crucial class of polymers that can be synthesized through various methods, integrating the properties of their constituent blocks into the final material. The importance of block copolymers can be highlighted in many areas of daily life by use of BCPs in practical applications in all fields. Block copolymers are generally used as composite materials in medicine, chemistry, nanotechnology, physics, and many other fields [11]. The sequential addition of the monomer(s), is the most commonly used technique that is successfully utilized to prepare block copolymers by using the monofunctional initiation system to form AB–, ABC–, and ABA–types [12]. Telechelic polymers are defined as macromolecules with reactive end groups and can be generally grouped as homo- and heterobifunctional telechelic polymers. Homobifunctional telechelics, synthesized using a bifunctional initiator after chain end modification, have the same functional group at both chain ends. Postpolymerization chemical modification of chain end of polyester diols to various functional groups (bromoester, xanthate etc.) is then utilized as a macroinitiator in the preparation of copolymers by CRPs.

Over the last decades, the preparation of block copolymers (BCPs) by controlled/living radical polymerization (CRP) techniques, such as reversible addition–fragmentation chain transfer (RAFT), atom radical transfer polymerization (ATRP), and nitroxide-mediated polymerization (NMP), has been studied in numerous publications, and evaluated in the fields of environmental, biomedical and pharmaceutical applications. Among the CRP techniques, RAFT polymerization is a robust method that replicates the characteristics of living polymerization. It allows the synthesis of block copolymers with a narrow molecular weight distribution and controlled architecture, enabling the polymerization of a wide range of functional monomers that are often incompatible with other CRP methods [13,14]. This polymerization suggests that an initiator produces radicals by thermal decomposition and a CTA provides controllability of polymerization through RAFT equilibrium [15]. The choice of the initial RAFT agents, with their effectiveness determined by the Z group (the activating group) and R group (the homolytic leaving group), is crucial to achieving good control of polymerization [16]. PCL macroCTAs can prepare the modification of PCL end groups, most commonly using xanthate and trithiocarbonate derivatives

Many studies have reported RAFT polymerization of the most well-known and studied hydrophilic monomers, including *N*-isopropylacrylamide (NIPAM), *N*-vinylpyrrolidone (NVP), *N*,*N*-dimethylacrylamide (DMA), and 2-(dimethylamino)ethyl methacrylate (DMAEMA), using PCL-based macro-CTAs. Poly(*N*-isopropylacrylamide) (PNIPAM) is a hydrophilic polymer, commonly employed to create temperature-responsive copolymers, when combined with other polymers [17]. Poly(*N*-vinyl pyrrolidone) (PNVP) is a hydrophilic polymer known for its applications in the pharmaceutical and biomedical fields, particularly in pharmaceutical tablets and hydrogels [18], owing to its characteristics such as water solubility and low toxicity [19]. In addition, poly(*N*,*N*-dimethylacrylamide) (PDMA), is a biocompatible hydrophilic polymer, and PDMA-based linear polymers, hydrogels [20], and blends, have found various applications in many fields, such as medicine, pharmacy, and molecular biology [21]. Advancements in synthesis resulting from the combination of CRP methods have enabled the preparation of a wide variety of block copolymers, consisting of a hydrophobic part containing PCL and a hydrophilic part that can include one or more of PNIPAM, PNVP, or PDMA.

Studies on block copolymers incorporating PCL and hydrophilic polymers, synthesized via a combination of ROP and RAFT polymerization, have gained significant attention. These copolymers utilize macro-CTAs obtained by attaching thiocarbonylthio derivatives [22], such as xanthates, trithiocarbonates, and dithiocarbamates, to the PCL ends; they have been extensively conducted and have attracted significant attention. PCL macroCTAs with xanthate terminated groups can be obtained through a two-step process of esterification and ionic substitution, from hydroxyl ended PCL, whereas trihiocarbonate- coupled PCL is prepared in a single step, Steglich esterification. Amphiphilic AB-type Poly(*ε*-caprolactone)-*b*-poly(*N*-vinylcaprolactam) (PCL-*b*-PVCL) [23] poly (*ε*-caprolactone)-*b*-poly (*N*-vinylpyrrolidone) (PCL-*b*-PNVP) [24,25], poly (*ε*-caprolactone)-*b*-poly (*N*-isopropylacrylamide) [26], ABA-type poly(*N*-isopropylacrylamide)-*b*-poly(*ε*-caprolactone)-*b*-poly(*N*-isopropylacrylamide) [27], and ABC-type [28] block copolymers were obtained through RAFT polymerization of various monomers using xanthate terminated PCL macroCTAs, prepared by reacting PCL-OH with 2-bromopropionyl bromide, followed by potassium O-ethyl xanthate (KEX). Another approach for obtaining PCL macroCTA involves the use of a widely utilized trithiocarbonate-based with an alkyl Z-group, 2-(dodecylthiocarbonothioylthio)-2-methylpropionic acid (DDMAT). With this approach, PCL macroCTAs are synthesized using alcohol derivate initiator via ROP and Steglich esterification of hydroxyl-terminated PCL with α-carboxyl capped DDMAT in the presence of *N*,*N*′-dicyclohexylcarbodiimide (DCC) and 4-(dimethylamino)pyridine (DMAP) [29]. Using trithio-carbonate-terminated PCL with DDMAT as macro-RAFT agents, AB-type [30], (AB)_4_-type [29], ABA-type [31], and ABC-type [32] block copolymers were prepared via RAFT polymerization of various hydrophilic monomers.

In recent years, the application areas of newly developed materials has gained significant importance. In particular, the unique properties of polymer materials have drawn considerable attention in various fields. As a result, the sensor applications of polymers with catalytic or conductive properties have emerged as a subject of growing interest. In addition, novel generation polymer sensors are preferred in the determination of phenolic compounds, which are an important class for human health. Therefore, a new application area of triblock copolymers was investigated for the determination of 4-hydroxy-3,5-dimethoxybenzoic acid (HDMBA), known as syringic acid, with an electrochemical technique using the proposed polymer nanosensors.

This study addresses two main research objectives. The first is to explore the usability of the novel synthesized initiator in the ROP of *ε*-CL and to utilize the novel PCL-based macro-CTAs obtained through modification in the RAFT polymerization of various monomers for the synthesis of block copolymers. The second objective is to investigate the potential application of the synthesized block copolymers in the determination of syringic acid, a target analyte that, to the best of the author’s knowledge, has not been previously studied using polymer-based systems. For this goal, a novel initiator, not previously used in polymerization of lactones, was chosen to obtain a middle part block copolymer, PCL diol, via ROP of *ε*-CL, and the modification of PCL ends with xanthate and trihiocarbonate groups was designed. ROP polymerization was carried out using a similar technique to that described in a previous study [12], with Sn(Oct)_2_ as a catalyst and an initiator similar to the synthesized and used diol initiator (MXTOH). In the next stage, using these novel PCL macroCTAs, a total of four triblock copolymers were synthesized through RAFT polymerization of various hydrophilic monomers, i.e., NIPAM, NVP, DMA, and DMAEMA, three of which were ABA- and one was ABC-type. The sensor capabilities of the synthesized novel triblock copolymers were compared, and the determination of the most sensitive sensor among them was investigated for the detection of 4-hydroxy-3,5-dimethoxybenzoic acid (HDMBA), also known as syringic acid, which is an important compound in terms of food and human health, was measured, to determine the most sensitive sensor among those investigated.

## 2. Experimental

### 2.1. Materials

Unless otherwise specified, all reactions were carried out under an argon atmosphere using standard Schlenk techniques. *ε*-Caprolactone (*ε*-CL, ABCR GmbH & Co. KG (Karlsruhe, Germany), 99%) was purified by distilling under vacuum after drying it with calcium hydride and stored over activated molecular sieves (4 Å) in a refrigerator. *N*-isopropylacrylamide (NIPAM, Sigma-Aldrich (Darmstadt, Germany), 97%), was purified by recrystallization from *n*-hexane/toluene mixture and dried under vacuum. *N*-Vinyl pyrrolidone (*N*VP, Acros (Geel, Belgium), 98%) was dried over anhydrous magnesium sulfate, distilled under reduced pressure, and stored in a refrigerator. 2-(Dimethylamino)ethyl methacrylate (DMAEMA, Sigma-Aldrich (Darmstadt, Germany) 98%) and *N*,*N*-dimethyl acrylamide (DMA, Sigma-Aldrich (Darmstadt, Germany), 99%) were purified by passing through a column of basic alumina to remove the inhibitor, prior to use. 2,2′-Azobis(isobutyronitrile) (AIBN) was received from TCI (Eschborn, Germany, >98%). After recrystallization from methanol, it was stored at 4 °C. *α,α*′-Dibromo-*m*-xylene (Sigma-Aldrich (Darmstadt, Germany), 97%), 2-bromopropionyl bromide Sigma-Aldrich (Darmstadt, Germany), 97%), potassium ethylxanthate (TCI (Eschborn, Germany), >95%), 2-(dodecylthiocarbonothioylthio)-2-methylpropionic acid (DDMAT, TCI, (Eschborn, Germany), >98%), triethylamine (TEA, Sigma-Aldrich (Darmstadt, Germany), ≥99%), pyridine (Sigma-Aldrich (Darmstadt, Germany), ≥99%), tin(II) 2-ethylhexanoate (Sn(Oct)_2_, Sigma-Aldrich (Darmstadt, Germany), >92.5%), and 4-hydroxy-3,5-dimethoxybenzoic acid (syringic acid; HDMBA, Sigma-Aldrich, Darmstadt, Germany) were used as received. Dichloromethane (DCM, Sigma-Aldrich, Darmstadt, Germany) was distilled from calcium hydride before use. *N*,*N*-Dimethylformamide (DMF, Sigma-Aldrich, Darmstadt, Germany) was distilled from magnesium sulfate. Conventional procedures were used for purification of all solvents [33].

### 2.2. Measurement

Transmission IR spectra were recorded on a FTIR spectrophotometer (PerkinElmer 1600, Shelton, CT, USA) in the spectral range 4000–400 cm^−1^ with samples. ^1^H NMR spectra were recorded on a Varian Mercury 400 MHz (Agilent, Santa Clara, CA, USA) spectrometer with CDCl_3_ as solvent at ambient temperature. The number average molecular weight M_n_ (GPC) and molecular weight distribution (*Đ*) values of PCL and PCL macroCTAs were analyzed by GPC, using a Shimadzu LC-2050C LT GPC system with column (Shimadzu Shim-pack GPC 804, Tokyo, Japan), THF LC Column 300 × 8.0 mm) in THF calibrated with PMMA standards at 25 °C. THF (HPLC grade) was used as an eluent at a flow rate of 1.0 mL/min for GPC analyses.

### 2.3. Construction Sensor and Electrochemical Measurement

A Vertex One versatile potentiostat/galvanostat analyzer (IVIUM, Eindhoven, The Netherlands) was used for electrochemical measurements. This analyzer operates with a triple electrode system, as follows: (i) a working electrode based on polymer modified sensor, (ii) an Ag/AgCl (BASi (West Lafayette, IN, USA) MF-2052) as a reference electrode, and (iii) a counter electrode (Pt wire, BASi, MW-1032). Before fabricating each polymer-based sensor, the glassy carbon electrode (GCE) surface was first polished with alumina powder (size 58 Å, ~150 mesh) for about 2 min to obtain a clean and smooth surface. Then, 2.0 mg of novel homopolymer (MXPCLOH) and triblock copolymers (MXTP1 and MXTP2) were precisely weighed and suspended in 1.0 mL of *N*,*N*-dimethylformamide (DMF) solvent in an ultrasonic water bath at room temperature for approximately 1 h. Then, 10 µL of suspensions of each polymer were dropped onto the clean surface of GCE (BASi MF 2012, 3.0 mm diameter) and dried at 50 °C for approximately 15 min. Before taking electrochemical measurements, the surface of the developed polymer/GCE sensors was activated by cyclic voltammetry (CV).

The electrochemical determination of syringic acid, a natural phenolic compound that is used to treat various diseases such as cancer, neurological and liver damage, was carried out in the most sensitive pH medium (pH 2.0 Britton Robinson buffer solution). In differential pulse voltammetry (DPV), parameters such as accumulation time (tacc), accumulation potential (Eacc), scan rate (mV/s), pulse amplitude (ΔE), and step potential (ΔEs), which significantly trigger the peak signal and potential, were used as tacc = 30 s, Eacc = 0 mV, ΔE = 50 mV, and ΔEs = 5 mV, respectively. The calibration graph for syringic acid was created by standard addition to pH 2.0 BR buffer solutions with DPV on the most sensitive MXTP2 nanosensor. Then, the linear working range and limit of detection (LOD) values were calculated on a MXTP2 polymer sensor.

### 2.4. Synthesis of ROP Initiator (MXTOH)

*m*-Xylene-bis(1-hydroxy-3-thia-propane) (MXTOH) was synthesized by the reaction of *α*,*α*′-dibromo-*m*-xylene with 2-mercaptoethanol in the presence of Na(s) metal in ethanol, according to a previously published procedure [34]. Yield: 3.86 g, 79%, colorless oil; ^1^H NMR (400 MHz, CDCl_3_, δ): 7.33–7.14 (m, 4H, Ar**H**), 3.69 (s, 4H, S−C**H_2_**−Ar), 3.61 (q, 4H, S−CH_2_−C**H_2_**−OH), 2.74 (s, 2H, −O**H**), 2.58 (t, 4H, S−C**H_2_**−CH_2_−OH); FTIR (ATR): ῡ_max_, (cm^−1^): 3356 (–OH), 2947–2870 (–CH_2_ aliphatic).

### 2.5. Synthesis of MXPCLOH

**Scheme 1:**
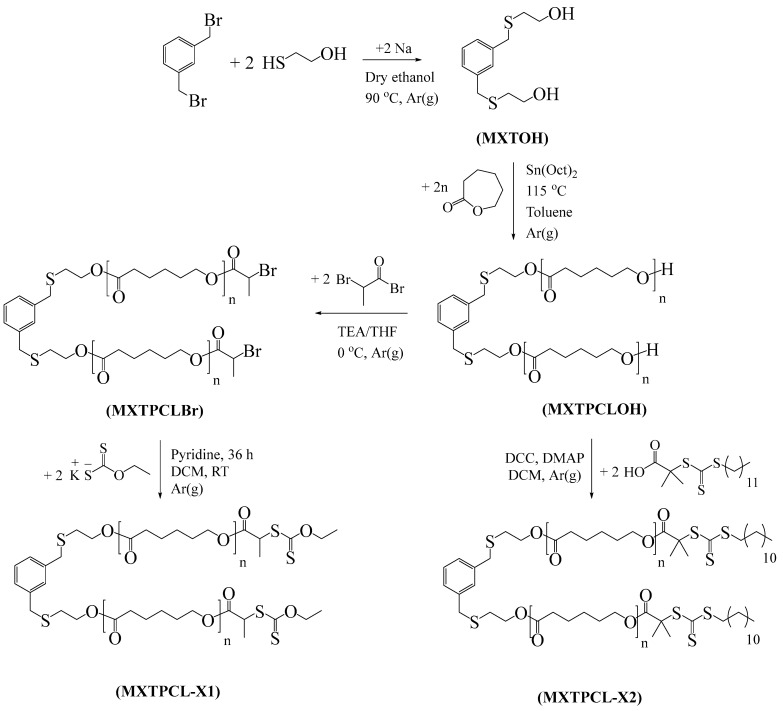
Synthesis of novel PCL-based macro chain agents.

The reaction mechanism is shown in Scheme 1. MXTPCLOH was synthesized via ROP of *ε*-CL with Sn(Oct)_2_ as a catalyst using MXTOH as the initiator with molar ratio of monomer to initiator, i.e., [M]:[I] = 20:1. Typically, MXTOH (0.258 g, 1 mmol), *ε*-CL (2.28 mL, 20 mmol), Sn(Oct)_2_ (34.2 µL, 0.15 mmol) and 1.5 mL toluene were introduced into a 25 mL Schlenk flask equipped with a magnetic stirrer. The reaction mixture underwent three pump-freeze-thaw cycles for degassing, followed by stirring and argon purging. The flask was placed in a preheated oil bath at 115 °C for 24 h. The crude polymer was dissolved in a small amount of dichloromethane (DCM) and then precipitated into an excess of cold methanol. The polymer was vacuum-dried at room temperature until a constant weight was achieved. The conversion (92%) was determined gravimetrically. The ^1^H NMR results found below are explained in the Results and Discussion section (Section 3.2).

M_n_(theo) = 2358 g/mol; M_n_(NMR) = 2450 g/mol; M_n_(GPC) = 4590 g/mol; *Ð* = 1.38; ^1^H NMR (400 MHz, CDCl_3_, δ): 7.33–7.14 (m, Ar**H**), 4.15 (t, S–CH_2_–C**H_2_**–O), 4.03 (t, CH_2_C**H_2_**–O–, in PCL), 3.71 (s, Ar–C**H_2_**–S–CH_2_–), 3.61 (t, –CH_2_C**H_2_**–OH, terminal in PCL), 2.61 (t, S–C**H_2_**–CH_2_–O), 2.28 (t, O=C–C**H_2_**CH_2_–, in PCL), 1.72–1.47 (m, –OCH_2_C**H_2_**CH_2_C**H_2_**CH_2_COO–in PCL), 1.47–1.27 (m, OCH_2_CH_2_C**H_2_**CH_2_CH_2_COO–, in PCL) ppm; FT–IR (ATR): ῡ_max_ (cm^−1^) = 3449 (–OH), 2943–2864 (aliphatic –CH_2_), 1724 (–C=O), 1293 (–C–O and –C–C), 1240 (asymmetric –C–O–C), 1180 (symmetric –C–O–C).

### 2.6. Synthesis of MXPCLBr

MXTPCLOH was converted to dibromo-ester functionalized MXTPCLBr by using 2-bromopropionyl bromide (Scheme 1). MXTPCLOH (1 g, 0.41 mmol, M_n_(NMR): 2450 g/mol) and TEA (0.28 mL, 1.04 mmol) were charged into a two-necked round-bottom flask and dissolved in dry DCM (20 mL) while stirring under argon atmosphere. The reaction was cooled down to 0 °C and 2-bromopropionyl bromide (0.19 mL, 1.64 mmol) in dry DCM (5 mL) was added dropwise to the cooled reaction mixture for 0.5 h. The reaction mixture was stirred at room temperature for 48 h. After the removal of precipitated salt, the filtrate was diluted with 25 mL of DCM and washed with 5% aqueous NaHCO_3_ (3 × 20 mL), then water (3 × 20 mL), dried over MgSO_4_, and filtered. The concentrated solution was precipitated into cold methanol and MXTPCLBr was dried under vacuum for 36 h.

M_n_(NMR) = 2925 g/mol; M_n_(GPC)= 5180 g/mol; *Ð* = 1.46; ^1^H NMR (400 MHz, CDCl_3_, δ): 7.37–7.15 (m, Ar**H**), 4.36 (q, –C**H**(CH_3_)Br), 4.22–4.10 (m, CH_2_C**H_2_**-OH, terminal in PCL S–CH_2_–C**H_2_**–O), 4.05 (t, CH_2_C**H_2_**–O–, in PCL), 3.73 (s, Ar–C**H_2_**–S–CH_2_–), 2.63 (t, S–C**H_2_**–CH_2_–O), 2.30 (t, O=C–C**H_2_**CH_2_–, in PCL), 1.81 (d, –CH(C**H_3_**)Br), 1.78–1.50 (m, –OCH_2_C**H_2_**CH_2_C**H_2_**CH_2_COO–, in PCL), 1.50–1.24 (m, OCH_2_CH_2_C**H_2_**CH_2_CH_2_COO–, in PCL) ppm.

### 2.7. Synthesis of Dixanthate Terminated PCL-Based Macro-RAFT CTA (MXTPCL-X1)

The functional polyester (MXTPCLBr) was converted into a PCL-based RAFT-CTA (MXTPCL-X1) via substitution reaction of the bromine end with KEX using a molar ratio of reagents; [(MXTPCLBr): KEX: pyridine]: 1:6:110; according to the procedure in the published literature [24,35,36]. In a typical process, MXTPCLBr (0.2 g, 0.068 mmol, M_n_(NMR): 2925 g/mol) and potassium ethylxanthate (KEX) (0.066 g, 0.408 mmol) were taken in a dried and argon purged round-bottom flask, and the flask was immersed in a cold ice bath. In another dried flask, pyridine (0.6 mL, 7.48 mmol) was dissolved in 20 mL DCM and this solution was added dropwise to the first reaction mixture during stirring for 30 min. The reaction mixture was maintained at room temperature for 48 h with stirring. Then, the mixture was diluted with 60 mL of DCM and washed successively with saturated NH_4_Cl solution (3 × 30 mL), saturated NaHCO_3_ solution (3 × 30 mL), and water (3 × 50 mL), dried over MgSO_4_ and filtered off. After the filtration was brought to dryness, the residue was dissolved in THF and precipitated into *n*-hexane. The PCL macro-CTA (MXTPCL-X1) was dried under vacuum.

M_n_(NMR) = 2740 g/mol; M_n_(GPC) = 4910 g/mol; *Ð* = 1.29; ^1^H NMR (400 MHz, CDCl_3_, δ): 7.34–7.16 (m, Ar**H**), 4.62 (q, O–C**H_2_**CH_3_), 4.37 (q, –C**H**(CH_3_)), 4.20–4.10 (m, CH_2_C**H_2_**–OH, terminal in PCL), 4.13 (S–CH_2_–C**H_2_**–O), 4.05 (t, CH_2_C**H_2_**–O–, in PCL), 3.73 (s, Ar–C**H_2_**-S–CH_2_–), 2.62 (t, S–C**H_2_**–CH_2_–O), 2.29 (t, O=C–C**H_2_**CH_2_–, in PCL), 1.75–1.50 (m, –OCH_2_C**H_2_**CH_2_C**H_2_**CH_2_COO–, in PCL), 1.56 (d, –CH(C**H_3_**)), 1.50–1.24 (m, OCH_2_CH_2_C**H_2_**CH_2_CH_2_COO–, in PCL, O–CH_2_C**H_3_**) ppm.

### 2.8. Synthesis of Trithiocarbonate-Capped PCL-Based Macro-RAFT CTA (MXTPCL-X2)

MXTPCL-X2 was prepared via an esterification reaction between MXTPCLOH and DDMAT using DCC/DMAP coupling agents, using the procedure previously reported in the literature [37]. MXTPCLOH (0.100 g, 0.041 mmol, M_n_(NMR): 2450 g/mol), DDMAT (0.150 g, 0.41 mmol), DCC (0.085 g, 0.41 mmol) and DMAP (4.9 mg, 0.041 mmol) were mixed in a Schlenk flask with 5 mL of DCM and the reaction mixture was stirred at room temperature for 3 d under argon atmosphere. After the removal of the reaction byproduct, dicyclohexylurea (DCU), the filtrate was evaporated and precipitated into cold diethyl ether. The PCL macro-CTA (MXTPCL-X2) was dried under vacuum.

M_n_(NMR) = 2850 g/mol; M_n_(GPC) = 5100 g/mol; *Ð* = 1.47; ^1^H NMR (400 MHz, CDCl_3_, δ): 7.31–7.17 (m, Ar**H**), 4.18 (t, S–CH_2_–C**H_2_**–O), 4.05 (t, CH_2_C**H_2_**–O–, in PCL), 3.73 (s, Ar–C**H_2_**–S–CH_2_–), 3.46 (br,t, –CH_2_C**H_2_**–O, terminal in PCL), 3.26 (t, SC**H_2_**(CH_2_)_10_ CH_3_), 2.63 (t, S–C**H_2_**–CH_2_–O), 2.30 (t, O=C–C**H_2_**CH_2_–, in PCL), 1.76–1.53 (m, –OCH_2_C**H_2_**CH_2_C**H_2_**CH_2_COO–,in PCL, (C**H_3_**)_2_CS), 1.50–1.21 (m, OCH_2_CH_2_C**H_2_**CH_2_CH_2_COO–, in PCL, SCH_2_(C**H_2_**)_10_ CH_3_), 0.87 (t, SCH_2_(CH_2_)_10_C**H_3_**) ppm.

### 2.9. Synthesis of Block Copolymers Using MXTPCL-X1 as macroCTA (MXTP1 and MXTP2)

**Scheme 2:**
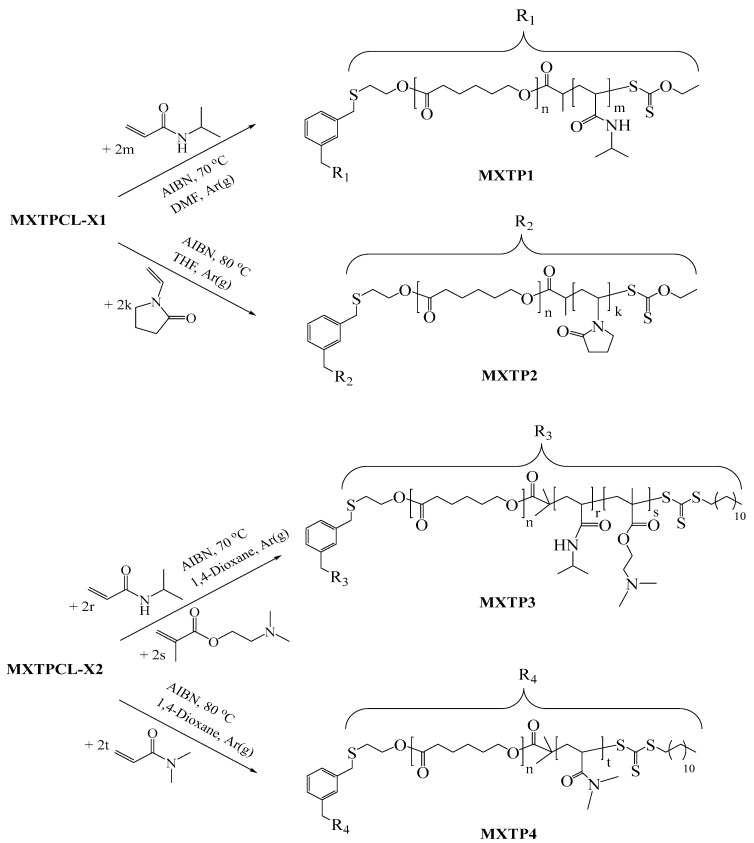
Synthesis of novel PCL-based block copolymers.

The syntheses of block copolymers was accomplished by RAFT of NIPAM and NVP using the MXTP-X1 as the PCL-based macroCTA at an ambient temperatures suitable for the type-specific polymerization of monomers (Scheme 2). MXTP1 was synthesized by RAFT with MXTP-X1 as the macro-RAFT agent [27]. MXTP-X1 (0.04 g, 0.0146 mmol, M_n_(NMR): 2740 g/mol), was dissolved with 3 mL of DMF in an argon-purged Schlenk tube, and NIPAM (0.170 g, 1.46 mmol) and AIBN (0.48 mg, 2.92 µmol) were then added. After three freeze-pump-thaw cycles, the flask was immersed in an oil bath at 70 °C and polymerization was allowed to proceed for 12 h. The polymerization mixture was diluted with THF (5 mL) and precipitated into 200 mL of *n*-hexane. The obtained polymer was purified by a dissolution/precipitation procedure twice. The resulting copolymer was separated by centrifugation and dried under vacuum for 24 h. The yield (40%) was determined gravimetrically.

MXTP2 was also prepared by RAFT with MXTP-X1 as the macro-RAFT agent. In a dried Schlenk flask, MXTP-X1 (0.045 g, 0.0164 mmol and M_n_(NMR): 2740 g/mol) were dissolved in 2 mL of THF. NVP (0.186 g, 0.179 mL, 1.64 mmol) and AIBN (0.54 mg, 3.28 µmol) were then added. The homogeneous solution was degassed with argon for 0.5 h with stirring. The flask was immersed in an oil bath at 80 °C and polymerization was allowed to proceed for 24 h. The polymerization mixture was diluted with 5 mL of THF, precipitated into 200 mL of *n*-hexane, and subsequently purified twice using a dissolution/precipitation process. MXTP2 was separated by centrifugation and dried under vacuum. Since the MXTP2 block copolymer may contain PCL and PNVP homopolymers, it was dispersed in selective solvents, ethyl acetate for PCL and water for PNVP, to remove these homopolymer impurities.

This purification process was performed separately for each homopolymer using the approach of collection by centrifugation and drying, in accordance with that reported in the literature [24]. After this procedure, the MXTP2 was redissolved with THF and precipitated into *n*-hexane. The resulting copolymer was separated by centrifugation and dried under vacuum for 48 h. The yield (37%) was determined gravimetrically.

### 2.10. Synthesis of Block Copolymers Using MXTPCL-X2 as macroCTA (MXTP3 and MXTP4)

The synthesis of ABC-type triblock copolymer, MXTP3, was performed by RAFT of NIPAM and DMAEMA using the MXTP-X2 as macroCTA [32]. Briefly, MXTP-X2 (0.02 g, 0.007 mmol, M_n_(NMR): 2850 g/mol), NIPAM (0.082 g, 0.70 mmol), and DMAEMA (0.022 g, 0.024 mL, 0.14 mmol) were dissolved in 2 mL of 1,4-dioxane, and AIBN (0.29 mg, 1.75 µmol) was then added. After three freeze-pump-thaw cycles, the flask was immersed in a preheated oil bath at 70 °C and polymerization was performed for 12 h. MXTP3 was obtained after precipitation in diethyl ether. The yield (62%) was determined gravimetrically.

The synthesis of ABA-type triblock copolymer, MXTP4, was also performed by RAFT of DMA with same macro-RAFT agent MXTP-X2 macroCTA [37]. Briefly, MXTP-X2 (0.02 g, 0.007 mmol, M_n_(NMR): 2850 g/mol) and DMA (0.21 g, 0.22 mL, 2.1 mmol) were mixed in 1 mL of 1,4-dioxane, and AIBN (0.29 mg, 1.75 µmol) was then added. After three freeze-pump-thaw cycles, the flask was immersed in a preheated oil bath at 70 °C and polymerization was performed for 5 h. MXTP4 was obtained after precipitation in diethyl ether and dried in a fume hood to obtain a bright yellow sticky polymer. The yield (91%) was determined gravimetrically.

## 3. Results and Discussion

### 3.1. Characterization of MXTPCLOH and MXTPCLBr

**Figure 1:**
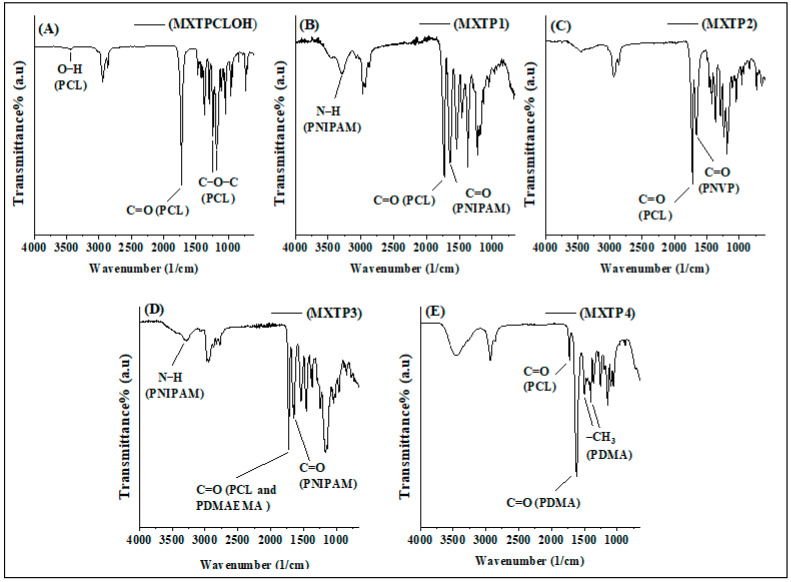
FT-IR spectra of PCL and block copolymers. (**A**) MXTPCLOH, (**B**) MXTP1, (**C**) MXTP2, (**D**) MXTP3, (**E**) MXTP4.

Novel PCL-based triblock copolymers were synthesized in four stages: (i) ROP of *ε*-CL, (ii) esterification of PCL-diol with 2-bromopropionyl bromide, (iii) synthesis of two novel PCL-based macro chain agents via substitution reaction and Steglich esterification, and (iv) preparation of ABA- and ABC-type triblock copolymers through RAFT polymerization. MXTPCLOH was synthesized via ROP of *ε*-CL with Sn(Oct)_2_ as a catalyst using MXTOH as the initiator with a molar ratio of monomer to initiator, i.e., [M]:[I] = 20:1, at 115 °C for 24 h. MXTPCLOH was characterized by FT-IR and ^1^H NMR. The FT-IR spectra of PXTPCLOH and the synthesized block copolymers are presented in Figure 1.

The FT-IR spectrum of MXTPCLOH is shown in Figure 1A. In the spectrum, the hydroxyl end group of PCL exhibited an absorption band that corresponded to the –O–H stretching at 3439 cm^−1^. The spectrum shows two bands at 2944 and 2866 cm^−1^, corresponding to symmetric and asymmetric CH_2_ stretching, respectively. The strong absorption peak at 1724 cm^−1^ belonging to the stretching vibration peak of C=O in the PCL chain in Figure 1A indicates the polymerization of *ε*-CL. Other characteristic bands of the PCL block are observed at 1293, 1240 and 1180 cm^−1^ belonging to stretching vibrations (–C–O and –C–C), (asymmetric –C–O–C), (symmetric –C–O–C), respectively. The band observed at 731 cm^−1^ is attributed to the CH_2_ rocking vibration of PCL which indicates linear aliphatic methylene of the PCL block [38].

**Figure 2:**
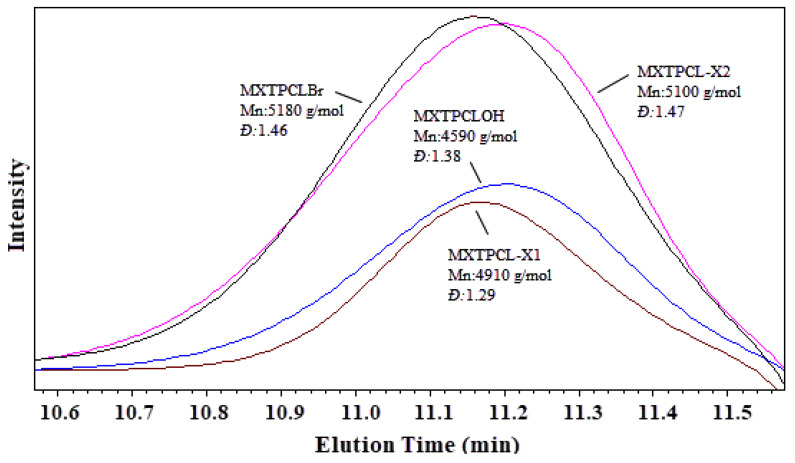
GPC traces of PCL (MXTPCLOH), PCL macroinitiator (MXTPCLBr), and PCL-macroCTAs (MXTPCL-X1 and MXTPCL-X2).

As seen in the ^1^H NMR spectrum (Supplementary Information Figure S1A) of PCL in CDCl_3_, the peak at 2.74 ppm corresponding to the primary –OH group proton of the initiator (MXTOH) disappears in comparison to the ^1^H NMR data spectrum of MXTOH given at the bottom of Section 2.4. The characteristic methylene protons peaks of the repeating unit of the PCL block are observed at 4.03 (H**^g^**), 2.28 (H**^d^**), 1.62 (H**^e^**), 1.35 (H**^f^**) ppm. The observation of methylene protons adjacent to primary hydroxymethylene end groups (–CH_2_C**H**_2_-OH, H**^g′^**) of PCL chain at 3.61 ppm indicates that the PCL block has a living character. The average molecular weight M_n_(NMR) of MXTPCLOH was calculated by comparing the peak integrals derived from the methylene protons “**g**” peak of PCL (δ = 4.03 ppm) and the methylene protons “**a**” peak of initiator (δ = 3.71 ppm) and is close to the theoretical number average molecular weight (M_n_(theo)). M_n_(GPC) and *Ð* were also determined as 4590 g/mol and 1.38. by GPC (Figure 2).

In the next step following the synthesis of MXTPCLOH, MXTPCLBr was prepared with chemical modification of the diol ends of MXTPCLOH via esterification by 2-bromopropionyl bromide. The bromo-ester functionalized macroinitiator (MXTPCLBr) was characterized by ^1^H NMR (see Supplementary Information Figure S1B). After esterification of the hydroxyl end groups of MXTPCLOH, two novel signals appear at 4.36 (quartet) and 1.81 (doublet) ppm, attributed to methine (H**^h^**) and methyl (H**^i^**) protons of the bromo propionate end groups, respectively [39]. The disappearance of the peak at 3.61 ppm, which corresponds to the methine protons adjacent to terminal hydroxyl end groups of the PCL end, and its overlap with multiple peaks’ methylene protons (S–CH_2_–C**H_2_**-O, H**^c^**) of MXTOH between 4.18 and 4.12 ppm, indicates that the esterification was successful [40]. M_n_(NMR) of MXTPCLBr was calculated by comparing the peak integrals derived from the methylene protons “**d**” of PCL (δ = 2.30 ppm) and the methine protons “**h**” of the bromo propionate end group (δ = 4.36 ppm) and was found to be 2925 g/mol. M_n_(GPC) and *Ð* were also determined as 5180 g/mol and 1.46 by GPC (Figure 2).

### 3.2. Characterization of MXTPCL-X1 and MXTPCL-X2

In this step of the study, the syntheses of two novel PCL-based macro-CTAs were designed by modification of the chain ends of PCL with xanthate and trithiocarbonate groups. The first PCL macro-CTA (MXTPCL-X1) was synthesized via substitution reaction of the bromine end of MXTPCLBr with KEX, as illustrated in Scheme 1. The ^1^H NMR spectrum of MXTPCL-X1 is displayed in the Supplementary Information (Figure S2A). Following the substitution reaction, a new signal at 4.62 ppm appears, corresponding to the methylene (H^j^) protons of the xanthate end group. The methyl (H**^k^**) protons of the xanthate end group could not be observed, since the peak of methylene protons at 1.37 ppm, corresponding to the PCL backbone, overlapped this peak. The peak at 1.81 ppm (Figure S1B), corresponding to the methyl (H**^i^**) protons of the bromo propionate end groups, shift to 1.56 ppm after the substitution reaction. M_n_(NMR) of MXTPCL-X1 was calculated by comparing the peak integrals derived from the methylene protons “**d**” of PCL (δ = 2.29 ppm) and the methylene protons of initiator “**b**” (δ = 2.62 ppm) and was found to be 2740 g/mol. M_n_(GPC) and *Ð* were also determined as 4910 g/mol and 1.29 by GPC (Figure 2).

The second PCL macro-RAFT agent (MXTPCL-X2) was prepared through PCL chain end-capping reaction esterification via Steglich esterification of the hydroxyl-ended MXTPCLOH with the carboxyl-capped DDMAT in the presence of DCC and DMAP as the coupling agent and catalyst, respectively. A representative ^1^H NMR of MXTPCL-X2 is shown in the Supplementary Information (Figure S2B). Compared with ^1^H NMR before (Figure S1A, in MXPCLOH], the signal of the primary hydroxymethylene end group (–CH_2_C**H**_2_-OH, H**^g’^**) of PCL chain at 3.61 ppm in MXTPCLOH shifts to 3.46 ppm after the esterification reaction, while the characteristic signals of the DDMAT end group appear at 3.26, 1.25 and 0.87 ppm, attributable to the methylene protons adjacent to the sulfur atom (−SC=SSC**H**_2_(CH_2_)_10_−, H**^m^**), aliphatic methylene protons adjacent to the methyl end group (−SC=SSCH_2_(C**H**_2_)_10_−, H**^n^**) and methyl protons of (−C**H**_3_, H**^p^**), respectively [29]. The methyl (H**^l^**) proton signal of the DDMAT end group could not be observed, due to overlap with the methylene protons at 1.64 ppm from the PCL backbone. M_n_(NMR) of MXTPCL-X2 was calculated by comparing the peak integrals derived from the methylene protons “**d**” of PCL (δ = 2.30 ppm) and the methylene protons “**m**” of the DDMAT end group (δ = 3.26 ppm) (Figure S2B) and found to be 2850 g/mol. M_n_(GPC) and *Ð* were also determined as 5100 g/mol and 1.47 by GPC (Figure 2).

### 3.3. Characterization of Block Copolymers

**Figure 3:**
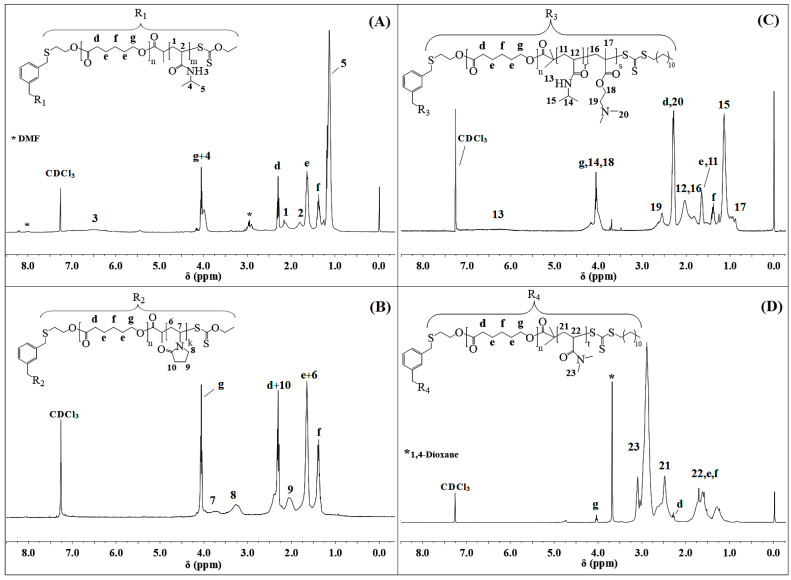
^1^H NMR spectra of block copolymers; (**A**) MXTP1, (**B**) MXTP2, (**C**) MXTP3, and (**D**) MXTP4.

The synthesis of block copolymers was accomplished by RAFT of various monomers using MXTP-X1 and MXTP-X2 as the PCL-based macro-CTAs at ambient temperatures. Using MXTP-X1 as the macro-RAFT agent, a PNIPAM-*b*-PCL-*b*-PNIPAM block copolymer (MXTP1) was prepared by RAFT of NIPAM, and the monomer MXTP1 was characterized by FTIR and ^1^H NMR. In the FTIR spectrum (Figure 1B), the appearance of the characteristic absorption peak of the carbonyl group at around 1726 cm^−1^, corresponding to PCL units, proves the existence of PCL segments in the block copolymer. The observation of the peaks at 3287 cm^−1^ (N-H stretching), 1639 cm^−1^, (C=O stretching) and 1539 cm^−1^, (N-H bending) belonging to the PNIPAM unit supports the formation of the block copolymer. The ^1^H NMR spectrum of MXTP1 (Figure 3A), shows that the resonance signals of the PNIPAM block, assigned to methine (H**^4^**) and methyl protons (H**^5^**), were noted at 3.99 and 1.13 ppm, respectively. In addition to these data of the PNIPAM block, a broad signal peak appears at 7.19−5.19 ppm (H**^3^**) belonging to the proton of the –NH group, and the signals at 2.15 (H**^1^**) and 1.80 (H**^2^**) ppm are also attributed to the methylene and methine group protons of the PNIPAM backbone, respectively. The appearance of the resonance signals of methylene protons of the PCL repeating unit at 4.09 (H**^g^**), 2.29 (H**^d^**), 1.63 (H**^e^**), and 1.37 (H**^f^**) ppm support the formation of the block copolymer [41]. M_n_(NMR) of MXTP1, was determined from ^1^H NMR (Figure 3A) by comparing the peak integrals derived from methyl protons of PNIPAM “**5**” (δ = 1.13 ppm) and overlapped the methylene “**g**” and methine protons “**4**” of PCL and PNIPAM (δ = 4.10−3.82 ppm), respectively. M_n_(NMR) was found to be 5554 g/mol.

P*N*VP-*b*-PCL-*b*-P*N*VP (MXTP2) was prepared by RAFT of NVP as a monomer using MXTP-X1 as the macro-RAFT agent and characterized by FTIR and ^1^H NMR. In the FTIR spectrum (Figure 1C), the characteristic absorption peaks of the carbonyl groups corresponding to PNVP and PCL units appear at 1660 and 1724 cm^−1^, respectively. The observation of an absorption band of the C–N group at 1289 cm^−1^ supports the evidence of PNVP incorporation into PCL blocks [42].

The ^1^H NMR spectrum of MXTP2 is displayed in Figure 3B. In the spectrum, methylene protons of the pyrrolidone ring, corresponding to the characteristic peaks of the PVP backbone, were detected at 3.59–3.09 (H**^8^**) and 2.17–1.90 (H**^9^**) ppm. The other signal of the methine proton (H**^7^**) of the PNVP chain, not overlapped with the PCL signals, appeared at 3.94–3.59 ppm. In addition, the characteristic methylene protons (H**^g^**) and (H**^f^**) of the PCL unit were observed at 4.06 and 1.38 ppm, respectively. The overlapped peaks at 2.58–2.17 ppm (H**^d+10^**) and 1.90–1.52 ppm (H**^e+6^**) belonging to the PNVP and PCL segments indicates the formation of the block copolymer [43]. M_n_(NMR) of MXTP2 was calculated by comparing the peak integrals derived from the methylene protons’ peaks of PNVP (δ = 3.59–3.09 ppm, peak “**8**”) and the methylene protons’ peak of PCL (δ = 4.06 ppm, peak “**g**”) in Figure S3. M_n_(NMR) was found to be 4158 g/mol (Supplementary Information Figure S3).

The third triblock copolymer, the ABC-type (MXTP3), was prepared by RAFT of NIPAM and DMAEMA as monomers using MXTP-X2 as the macro-RAFT agent and characterized by FTIR and ^1^H NMR. In the FTIR spectrum (Figure 1D), the appearance of the absorption peak of the carbonyl group at around 1725 cm^−1^, corresponding to the overlapped PCL and PDMAEMA units, proves the existence of PDMAEMA segments in the block copolymer. The observation of the peaks at 3289 cm^−1^ (N-H stretching) and 1631 cm^−1^ (C=O stretching) belonging to the PNIPAM unit supports the extension of the PCL chain with PNIPAM and PDMAEMA blocks in the block copolymer [44].

The ^1^H NMR spectrum of MXTP3 displayed the characteristic peaks of PNIPAM and PDMAEMA blocks as well as PCL block (Figure 3C). With no overlaps, the characteristic peaks of PNIPAM, PDMAEMA and PCL belonging to the proton (H**^13^**) of the –NH group, methylene protons (H**^19^**) of PDMAEMA and methylene protons (H**^f^**) of the PCL units, appear at 7.14−5.66, 2.78–2.42 and 1.38 ppm, respectively. The resonance signal of the PNIPAM block, assigned to methyl protons (H**^15^**), was noted at 1.13 ppm. In the spectrum, it is observed that the peaks of all three components of the copolymer overlap between 4.37–3.83 ppm (H**^g+14+18^**). Other overlapping peaks are also observed at 2.41–2.20 (H**^d+20^**), 2.20–1.72 (H**^12+16^**), and 1.73–1.44 (H**^e+11^**), belonging to PCL and the PDMAEMA, PNIPAM and PDMAEMA backbone, and PCL and PNIPAM, respectively. M_n_(NMR) of MXTP3 was calculated by comparing the proton signals at δ = 1.13 ppm (peak “**15**”), δ = 4.06 ppm (peak “**g**”) and at δ = 4.37–3.83 ppm (“**g+14+18**”), ascribed to the methyl group of PNIPAM, the methylene group of PCL, and the overlapping peak, which also includes the PDMAEMA block, respectively. M_n_(NMR) was found at to be 7800 g/mol.

The fourth block copolymer (MXTP4) was synthesized by RAFT of DMA as the monomer using MXTP-X2 as the macro-RAFT agent. MXTP4 was characterized by FTIR and ^1^H NMR. In the FTIR spectrum (Figure 1E), the absorption peak at 1622 cm^−1^ corresponds to the amide carbonyl group of the PDMA units. In addition to the observation of a carbonyl group of PCL at 1722 cm^−1^, asymmetric and symmetric –CH_3_ deformation appear at 1496 and 1398 cm^−1^, respectively. The disappearance of the band related to the C=C group at 982 cm^−1^, corresponding to the DMA monomer, show that the polymerization of DMA has been performed successfully [20,45]. The ^1^H NMR spectrum of MXTP4 is shown in Figure 3D. In the spectrum, methyl protons related to PDMA units were detected at 3.22–2.72 (H**^23^**) ppm. The signal of the methine proton (H**^21^**) related to the PDMA chain appeared at 2.72–2.22 ppm and overlapped with the methylene protons (H**^d^**) of the PCL unit. In addition, the characteristic methylene protons (H**^g^**) at 4.03 ppm and the overlapped peaks at 1.92–0.99 ppm (H**^e+f+22^**), belonging to PNVP and PCL segments, indicates the formation of the block copolymer [45]. M_n_(NMR) of MXTP4 was calculated by comparing the peak integrals derived from the methyl protons’ peak of PDMA (δ = 3.22–2.72 ppm, peak “**23**”) and the methylene protons’ peak of PCL (δ = 4.03 ppm, peak “**g**”) (in Figure S4). M_n_(NMR) was calculated as 32,290 g/mol (Supplementary Information Figure S4).

### 3.4. Sensor Ability

Phenolic compounds are the best-known electroactive substances [46,47]. The phenol in these substances is formed by oxidation of the functional structure [48]. The determination of such substances is of great importance, especially since they are used in antioxidant, antimicrobial, and cancer treatments [46,49]. 4-Hydroxy-3,5-dimethoxybenzoic acid (HDMBA), also known as syringic acid (C_9_H_10_O_5_), is a naturally occurring phenolic compound that is abundantly present in various foods, including olives, dates, spices, açai, honey, wine, and vinegar. In addition, HDMBA has unique properties such as anti-angiogenic, antioxidant, antimicrobial, and anti-inflammatory [50].

**Figure 4:**
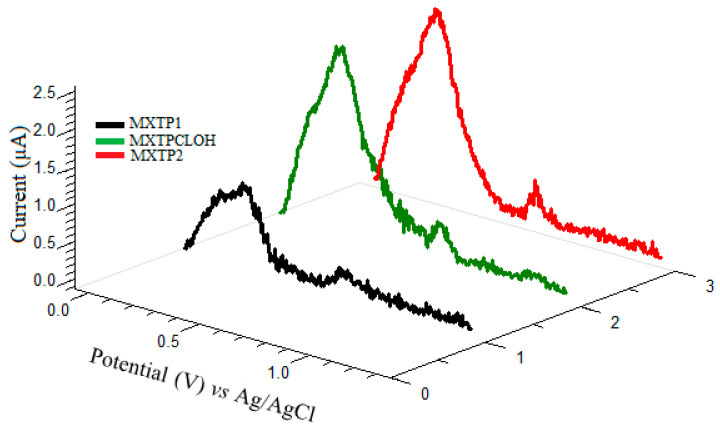
Comparison of novel developed polymer based nanosensors for the determination of syringic acid.

Polymer materials are used in sensor applications due to their two unique properties. The first of these properties is a conductive property and the other is catalytic performance. These unique properties exhibited by polymers have contributed to the development of highly sensitive, extremely selective, and long-life electrochemical sensors with synergistic effects. Furthermore, electrochemical measurements were obtained to determine the sensor capacities of synthesized homo polymer and triblock copolymers. Also, the sensor capabilities of the synthesized novel triblock copolymers were compared, and the determination of the most sensitive sensor among them was investigated for the detection of 4-hydroxy-3,5-dimethoxybenzoic acid (HDMBA), also known as syringic acid, which is an important compound in terms of food and human health. Firstly, the sensitivities of the novel PCL homo polymer and two triblock copolymer sensors were examined with the DPV electrochemical technique in the most ideal pH environment (pH 2.0) for the determination of syringic acid (Figure 4). For the anodic peak of 2 µg/mL syringic acid, approximately 0.7 V was obtained with MXTPCLOH, while a potential close to 0.8 V was obtained with the other copolymers. This indicates that MXTPCLOH exhibits a more active catalytic effect compared to MXTP1 and MXTP2. However, when the sensitivities of the sensors were examined, MXTP2 increased the anodic signal of syringic acid by approximately 1.8 times compared to the MXTP1 and MXTPCLOH sensors. This phenomenon can be explained in two ways. First, the MXTP2 sensor provides a larger surface area, making it more sensitive for the determination of syringic acid. The second explanation is more complex and is related to the sensor affinity of the analyte. This process facilitates the transport of the analyte to the electrode surface, as evidenced by the adsorption properties of the sensor. Consequently, this enhances the transport of the HDMBA analyte to the electrode surface. As a result, in both cases, the MXTP2 polymer-based sensor demonstrates higher sensitivity for the determination of syringic acid.

**Figure 5:**
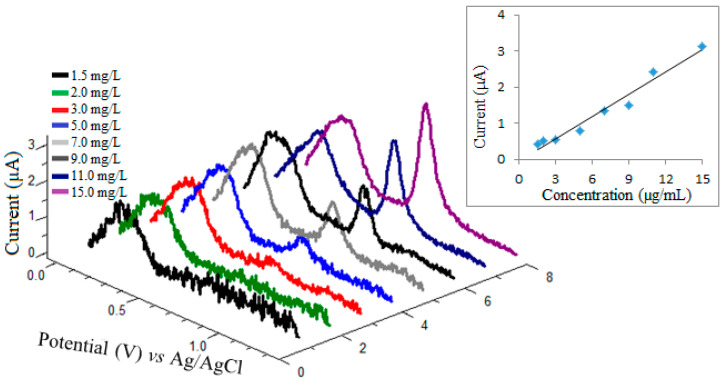
DP voltammograms at different concentrations on the MXTP2 polymer sensor developed for the determination of syringic acid.

The calibration graph of the parameters (pH, frequency, pulse amplitude, etc.) included in the DPV technique was created on the MXTP2 sensor by a standard addition method in syringic acid solution under ideal conditions (Figure 5). A linear working range (r = 0.9836) was obtained of between 1.5 µg/mL and 15.0 µg/mL concentrations of syringic acid.

*Ip* (µA) = 0.204 (µA mL/µg) (C) − 0.037 (µA) (R^2^ = 0.9675)

Based on the obtained graph for the determination of syringic acid, the limit of detection (LOD) value was calculated on the MXTP2 sensor by DVP. For this, the “3s/m” formula was used and “s” in this formula represents the standard deviation of the cut-off point of the calibration graph, and “m” represents the slope. When all the necessary numerical data were substituted into the formula, the LOD value was found to be 0.44 µg/mL. In conclusion, a novel, alternative and sensitive polymer-based sensor for the determination of syringic acid, an important natural phenolic compound, was successfully realized.

## 4. Conclusions

This study addresses two main research objectives. The first is to explore the usability of the novel synthesized initiator in the ROP of ε-CL and to utilize the novel PCL-based macro-CTAs obtained through modification in the RAFT polymerization of various monomers for the synthesis of block copolymers. The second objective is to investigate the potential application of the synthesized block copolymers in the determination of syringic acid, a target analyte that, to the best of the author’s knowledge, has not been previously studied using polymer-based systems. For this purpose, novel ABA-type triblock copolymers (PNIPAM-*b*-PCL-*b*-PNIPAM, PNVP-*b*-PCL-*b*-PNVP, and PDMA-*b*-PCL-*b*-PDMA) and an ABC-type copolymer (P(DMAEMA-*co*-NIPAM)-*b*-PCL-*b*-P(NIPAM-*co*-DMAEMA)) were successfully synthesized. The synthesis involved a combination of ROP and RAFT polymerization using bifunctional PCL-based RAFT macroinitiators with an *m*-xylene-bis(2-mercaptoethyloxy) core. A novel initiator (MXTOH), not previously used in polymerization of lactones and lactides via ROP, was designed to synthesize PCL, a hydrophobic polymer, in the middle part of the block copolymers. The novel PCL-based macroCTAs, MXTPCL-X1 and MXTPCL-X2, were prepared by substitution and esterification reactions with suitable PCL ends using potassium ethylxanthate (KEX) and 2-(dodecylthiocarbonothioylthio)-2-methylpropionic acid (DDMAT) as chain transfer agents, respectively. The preparations of ABA- and ABC-type triblock copolymers were performed successfully by RAFT polymerization of several hydrophilic monomers using PCL-based macro-CTAs. The sensor capabilities of the novel synthesized triblock copolymers were investigated using the determination of syringic acid, and it was determined that the most sensitive polymer sensor was MXTP2. The working range was between 1.5 µg/mL and 15 µg/mL and the limit of detection (LOD) was found to be 0.44 µg/mL using DPV on the MXTP2 polymer sensor for the analysis of syringic acid. Furthermore, the MXTPCLOH nanosensor had more active catalytic properties than MXTP1 and MXTP2, but MXTP2 also showed more conductive properties than the other (MXTP1 and MXTPCLOH) polymeric nanosensors at the first determination of the phenolic compound syringic acid.

## Data Availability

All data and materials produced from this study are publicly accessible.

## Supplementary Materials

The following supporting information can be downloaded at: https://www.mdpi.com/article/10.3390/polym17070873/s1, Figure S1: ^1^H NMR spectra of MXTPCLOH (A) and MXTPCLBr (B). Figure S2: ^1^H NMR spectra of PCL-based macro-CTAs; MXTPCL-X1 (A) and MXTPCL-X2 (B). Figure S3: ^1^H NMR spectrum of MXTP2 using MXTPCL-X1. Figure S4: ^1^H NMR spectrum of MXTP4 using MXTPCL-X2.

## References

[1] Ali Akbari Ghavimi S., Ebrahimzadeh M.H., Solati-Hashjin M., Abu Osman N.A. (2015). Polycaprolactone/starch composite: Fabrication, structure, properties, and applications. J. Biomed. Mater. Res. Part A.

[2] Archer E., Torretti M., Madbouly S. (2023). Biodegradable polycaprolactone (PCL) based polymer and composites. Phys. Sci. Rev..

[3] Mohamed R.M., Yusoh K. (2016). A review on the recent research of polycaprolactone (PCL). Adv. Mater. Res..

[4] Oney-Montalvo J.E., Dzib-Cauich D.A., de Jesús Ramírez-Rivera E., Cabal-Prieto A., Can-Herrera L.A. (2024). Applications of polycaprolactone in the food industry: A review. Czech J. Food Sci..

[5] Washington K.E., Kularatne R.N., Karmegam V., Biewer M.C., Stefan M.C. (2017). Recent advances in aliphatic polyesters for drug delivery applications. Wiley Interdiscip. Rev. Nanomed. Nanobiotechnol..

[6] Li Y., Chu Z., Li X., Ding X., Guo M., Zhao H., Yao J., Wang L., Cai Q., Fan Y. (2017). The effect of mechanical loads on the degradation of aliphatic biodegradable polyesters. Regen. Biomater..

[7] Seyednejad H., Ghassemi A.H., van Nostrum C.F., Vermonden T., Hennink W.E. (2011). Functional aliphatic polyesters for biomedical and pharmaceutical applications. J. Control. Release.

[8] Jérôme C., Lecomte P. (2008). Recent advances in the synthesis of aliphatic polyesters by ring-opening polymerization. Adv. Drug Deliv. Rev..

[9] Wang K., Ni M., Dundas A.A., Dimitrakis G., Irvine D.J. (2023). Ring opening polymerisation of ɛ-caprolactone with novel microwave magnetic heating and cyto-compatible catalyst. Front. Bioeng. Biotechnol..

[10] Kricheldorf H.R., Weidner S.M. (2021). ROP of L-lactide and ε-caprolactone catalyzed by tin (ii) and tin (iv) acetates–switching from COOH terminated linear chains to cycles. J. Polym. Sci..

[11] Politakos N., Avgeropoulos A. (2023). Advances and Applications of Block Copolymers. Polymers.

[12] Mısır M., Savaskan Yılmaz S., Bilgin A. (2023). Synthesis and Characterization of ABA-Type Triblock Copolymers Using Novel Bifunctional PS, PMMA, and PCL Macroinitiators Bearing p-xylene-bis (2-mercaptoethyloxy) Core. Polymers.

[13] Semsarilar M., Abetz V. (2021). Polymerizations by RAFT: Developments of the Technique and Its Application in the Synthesis of Tailored (Co) polymers. Macromol. Chem. Phys..

[14] Sofla S.F.I., Abbasian M., Mirzaei M. (2019). Synthesis and micellar characterization of novel pH-sensitive thiol-ended triblock copolymer via combination of RAFT and ROP processes. Int. J. Polym. Mater. Polym. Biomater..

[15] Lee Y., Boyer C., Kwon M.S. (2023). Photocontrolled RAFT polymerization: Past, present, and future. Chem. Soc. Rev..

[16] Moad G., Rizzardo E., Thang S.H. (2013). RAFT polymerization and some of its applications. Chem.—Asian J..

[17] Lee R.-S., Lin C.-H., Aljuffali I.A., Hu K.-Y., Fang J.-Y. (2015). Passive targeting of thermosensitive diblock copolymer micelles to the lungs: Synthesis and characterization of poly (N-isopropylacrylamide)-block-poly (ε-caprolactone). J. Nanobiotechnol..

[18] Liu X., Xu Y., Wu Z., Chen H. (2013). Poly (N-vinylpyrrolidone)-modified surfaces for biomedical applications. Macromol. Biosci..

[19] Franco P., De Marco I. (2020). The Use of Poly (N-vinyl pyrrolidone) in the Delivery of Drugs: A Review. Polymers.

[20] Bashir S., Hina M., Ramesh S., Ramesh K. (2021). Flexible and self-healable poly (N, N-dimethylacrylamide) hydrogels for supercapacitor prototype. Colloids Surf. A Physicochem. Eng. Asp..

[21] Algi M.P., Okay O. (2014). Highly stretchable self-healing poly (N, N-dimethylacrylamide) hydrogels. Eur. Polym. J..

[22] Roka N., Kokkorogianni O., Kontoes-Georgoudakis P., Choinopoulos I., Pitsikalis M. (2022). Recent advances in the synthesis of complex macromolecular architectures based on poly (n-vinyl pyrrolidone) and the RAFT polymerization technique. Polymers.

[23] Yu Y.C., Kang H.U., Youk J.H. (2012). Synthesis and micellar characterization of thermosensitive amphiphilic poly (ε-caprolactone)-b-poly (N-vinylcaprolactam) block copolymers. Colloid Polym. Sci..

[24] Mishra A.K., Patel V.K., Vishwakarma N.K., Biswas C.S., Raula M., Misra A., Mandal T.K., Ray B. (2011). Synthesis of well-defined amphiphilic poly (ε-caprolactone)-b-poly (N-vinylpyrrolidone) block copolymers via the combination of ROP and xanthate-mediated raft polymerization. Macromolecules.

[25] Mishra A.K., Ramesh K., Paira T.K., Srivastava D.N., Mandal T.K., Misra N., Ray B. (2013). Synthesis and self-assembly properties of well-defined four-arm star poly (ε-caprolactone)-b-poly (N-vinylpyrrolidone) amphiphilic block copolymers. Polym. Bull..

[26] de Moraes R.M., de Carvalho L.T., Teixeira A.J.R., Medeiros S.F., dos Santos A.M. (2023). Well-defined amphiphilic poly (ε-caprolactone)-b-poly (N-isopropylacrylamide) and thermosensitive micelles formulation. Iran. Polym. J..

[27] Mishra A.K., Vishwakarma N.K., Patel V.K., Biswas C.S., Paira T.K., Mandal T.K., Maiti P., Ray B. (2014). Synthesis, characterization, and solution behavior of well-defined double hydrophilic linear amphiphilic poly (N-isopropylacrylamide)-b-poly (ε-caprolactone)-b-poly (N-isopropylacrylamide) triblock copolymers. Colloid Polym. Sci..

[28] Peña J.A., Gutiérrez S.J., Villamil J.C., Agudelo N.A., Pérez L.D. (2016). Policaprolactone/polyvinylpyrrolidone/siloxane hybrid materials: Synthesis and in vitro delivery of diclofenac and biocompatibility with periodontal ligament fibroblasts. Mater. Sci. Eng. C.

[29] Bian Q., Xiao Y., Lang M. (2012). R-RAFT approach for the polymerization of N-isopropylacrylamide with a star poly (ε-caprolactone) core. J. Polym. Sci. Part A Polym. Chem..

[30] Sheng L., Zhu X., Sun M., Lan Z., Yang Y., Xin Y., Li Y. (2023). Tumor Microenvironment-Responsive Magnetic Nanofluid for Enhanced Tumor MRI and Tumor multi-treatments. Pharmaceuticals.

[31] Kim T., Mays J., Chung I. (2018). Porous poly (ε-caprolactone) microspheres via UV photodegradation of block copolymers prepared by RAFT polymerization. Polymer.

[32] Yuan W., Shen J., Zou H. (2015). Amphiphilic block copolymer terminated with pyrene group: From switchable CO_2_-temperature dual responses to tunable fluorescence. RSC Adv..

[33] Perrin D.D., Armarego W.L., Perrin D.R. (1988). Purification of Laboratory Chemicals.

[34] de Groot B., Loeb S.J., Shimizu G.K. (1994). Large-ring S6-thiacyclophanes as ditopic macrocycles. Synthesis and structures of 2, 5, 8, 17, 20, 23-hexathia [9.9]-o-cyclophane, HT [9.9] OC, 2, 5, 8, 17, 20, 23-hexathia [9.9]-m-cyclophane, HT [9.9] MC, and [Ag2 (CH3CN) 2 (HT [9.9] OC)][BF4] 2. Inorg. Chem..

[35] Mısır M., Azarkan S.Y. (2024). Synthesis, characterization, and cytotoxicity analyses of ABA-type block copolymer bearing p-xylene-bis (2-mercaptoethyloxy) core. J. New Results Sci..

[36] Mısır M. (2025). Synthesis, characterization and cytotoxicity analyzes of novel AB-Type Amphiphilic Block Copolymers. Health Sci. Q..

[37] Yu W., Foster J.C., Dove A.P., O’Reilly R.K. (2020). Length control of biodegradable fiber-like micelles via tuning solubility: A self-seeding crystallization-driven self-assembly of poly (ε-caprolactone)-containing triblock copolymers. Macromolecules.

[38] Li X., Zhang Q., Ye D., Zhang J., Guo Y., You R., Yan S., Li M., Qu J. (2017). Fabrication and characterization of electrospun PCL/Antheraea pernyi silk fibroin nanofibrous scaffolds. Polym. Eng. Sci..

[39] Banerjee S.L., Hoskins R., Swift T., Rimmer S., Singha N.K. (2018). A self-healable fluorescence active hydrogel based on ionic block copolymers prepared via ring opening polymerization and xanthate mediated RAFT polymerization. Polym. Chem..

[40] Lo Y.-L., Chen G.-J., Feng T.-H., Li M.-H., Wang L.-F. (2014). Synthesis and characterization of S-PCL-PDMAEMA for co-delivery of pDNA and DOX. RSC Adv..

[41] Liu X., Ma R., Shen J., Xu Y., An Y., Shi L. (2012). Controlled release of ionic drugs from complex micelles with charged channels. Biomacromolecules.

[42] Dhumale V.A., Gangwar R.K., Datar S.S., Sharma R.B. (2012). Reversible aggregation control of polyvinylpyrrolidone capped gold nanoparticles as a function of pH. Mater. Express.

[43] Debuigne A., Schoumacher M., Willet N., Riva R., Zhu X., Rütten S., Jérôme C., Detrembleur C. (2011). New functional poly (N-vinylpyrrolidone) based (co) polymers via photoinitiated cobalt-mediated radical polymerization. Chem. Commun..

[44] Motamedi S., Massoumi B., Jaymand M., Hamishehkar H. (2020). A dual stimuli-responsive star-shaped nanocarrier as de novo drug delivery system for chemotherapy of solid tumors. J. Polym. Res..

[45] Egghe T., Cools P., Van Guyse J.F., Asadian M., Khalenkow D., Nikiforov A., Declercq H., Skirtach A.G., Morent R., Hoogenboom R. (2019). Water-stable plasma-polymerized N, N-dimethylacrylamide coatings to control cellular adhesion. ACS Appl. Mater. Interfaces.

[46] Demir E., Silah H., Aydogdu N. (2021). Electrochemical applications for the antioxidant sensing in food samples such as citrus and its derivatives, soft drinks, Supplementary Food and Nutrients. Citrus-Research, Development and Biotechnology.

[47] Demır E. (2019). Sensitive and selective pathway of total antioxidant capacity in commercially lemon, watermelon and mango-pineapple cold teas by square wave adsorptive stripping voltammetry. Gazi Univ. J. Sci..

[48] Yildirim S., Gok I., Demir E., Tokusoglu O. (2022). Use of electrochemical techniques for determining the effect of brewing techniques (espresso, Turkish and filter coffee) and roasting levels on total antioxidant capacity of coffee beverage. J. Food Process. Preserv..

[49] Öztürk M., Demir E., Ozdal T. (2021). Voltammetric and spectrophotometric pathways for the determination of total antioxidant capacity in commercial turnip juice. J. Turk. Chem. Soc. Sect. A Chem..

[50] Bandyopadhyay D., Nag S., Das D., Roy R.B. (2024). Detection of syringic acid in food extracts using molecular imprinted polyacrylonitrile infused graphite electrode. J. Food Compos. Anal..

