# Supplementary material for: Synthesis, Characterization and Sensor Application of Novel PCL-Based Triblock Copolymers
Source: Polymers (Basel). 2025 Mar 25;17(7):873. doi: 10.3390/polym17070873 (PMC11990995; doi:10.3390/polym17070873)
Supplement: Supplementary file 1 [file polymers-17-00873-s001.zip › polymers-3488070-supplementary.pdf]

Supplementary Materials

This document contains supplementary materials supporting the main text, including NMR spectra of MXTPCLOH, MXTPCLBr and PCL-macroCTAs (MXTPCL-X1 and MXTPCL-X2), and also NMR calculations and spectra related to the molecular weight determination of the block copolymers (MXTP2 and MXTP4).

Supplementary Material

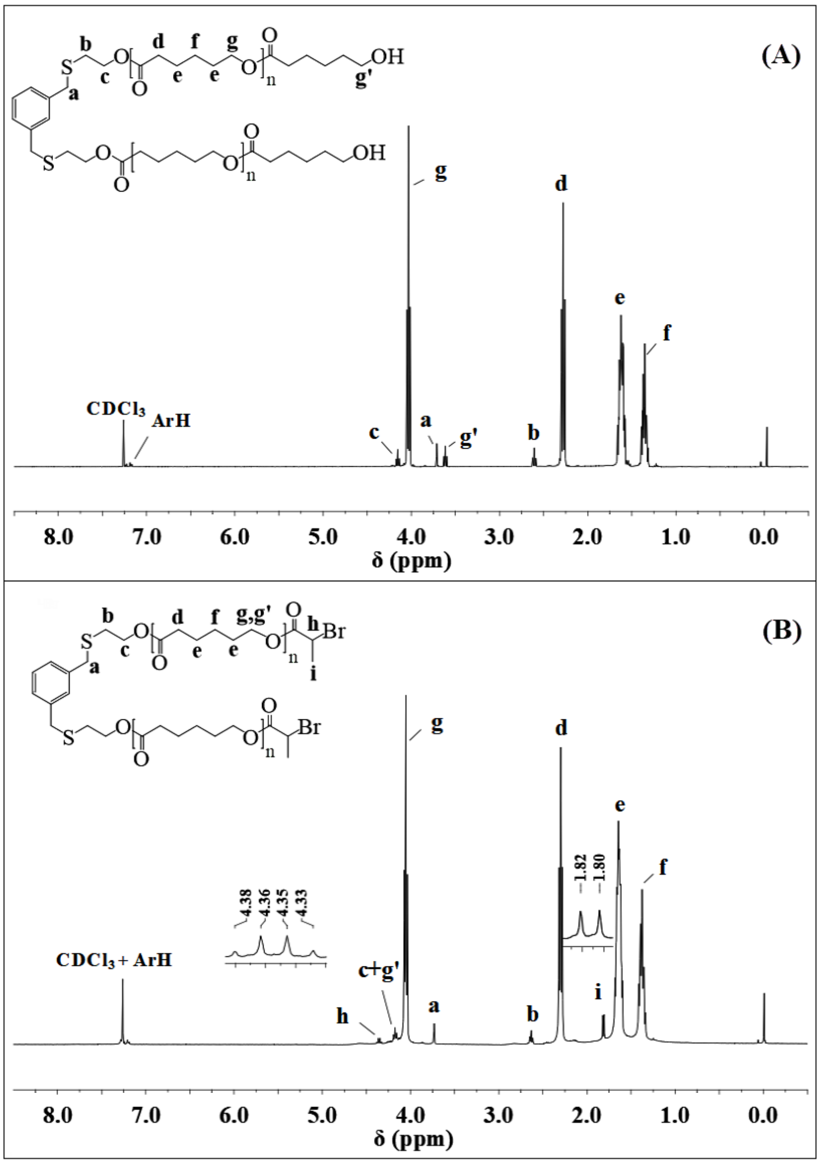

Figure S1. ^{1}H NMR spectra of MXTPCLOH (A) and MXTPCLBr (B).

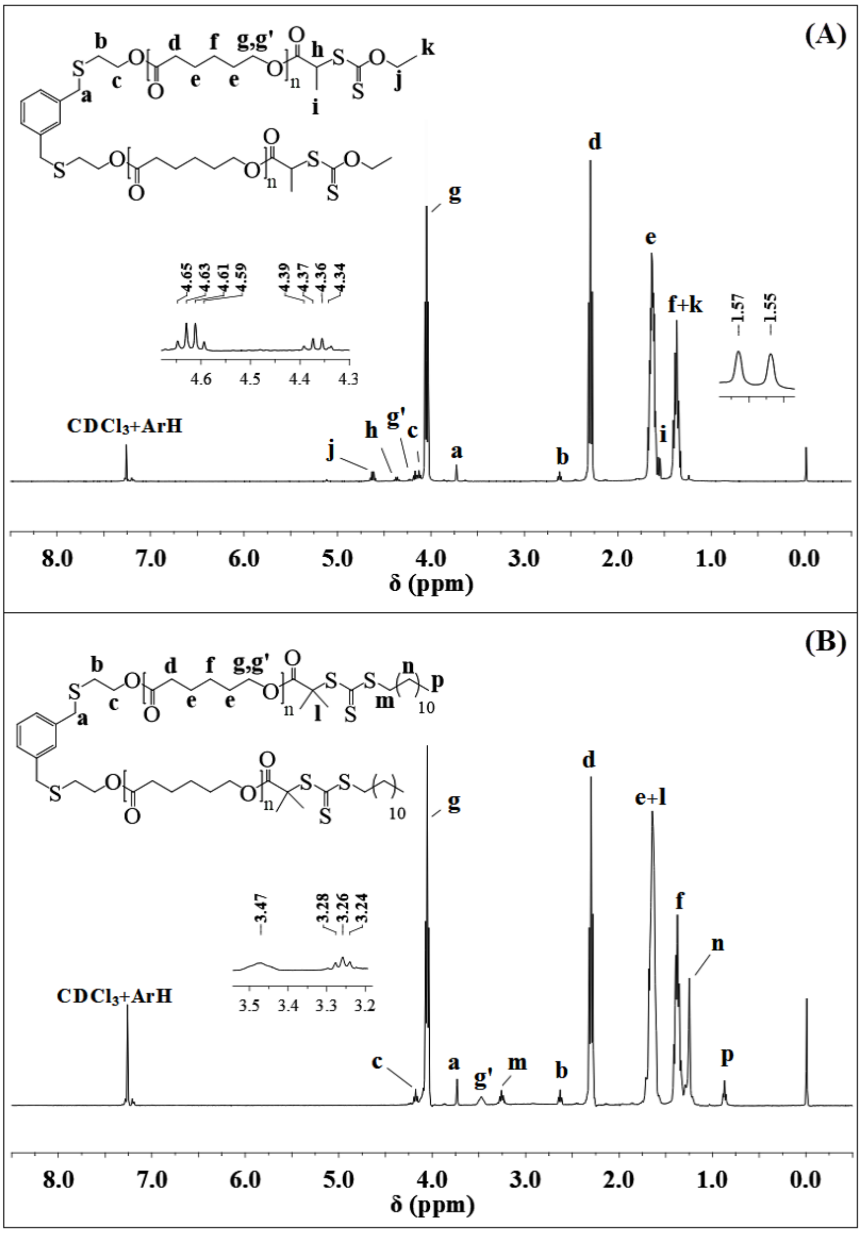

Figure S2. ^{1}H NMR spectra of PCL-based macro-CTAs; MXTPCL-X1 (A) and MXTPCL-X2 (B).

M_n(NMR) of MXTP2 was calculated by comparing the peak integrals derived from the methylene protons' peaks of PNVP (δ =3.59–3.09 ppm, peak "**8**") and the methylene protons' peak of PCL (δ = 4.06 ppm, peak "**g**") in Figure S3 according to (1) and (2)

$$M_n \text{ (NMR)}= DP_{PNVP}\ x\ M_{monomer} + M_{n,MXTPCL-X1} \quad (1)$$

$$DP_{PNVP} = \frac{I_8}{4H}\, x \frac{4H}{I_g}\, x\ DP_{PCL} \quad (2)$$

Here, DP_{PNVP} and DP_{PCL} are degrees of polymerization for PNVP and PCL segments, respectively. $M_{monomer}$ is also the molecular weight of the NVP. M_n(NMR) was calculated 4158 g/mol.

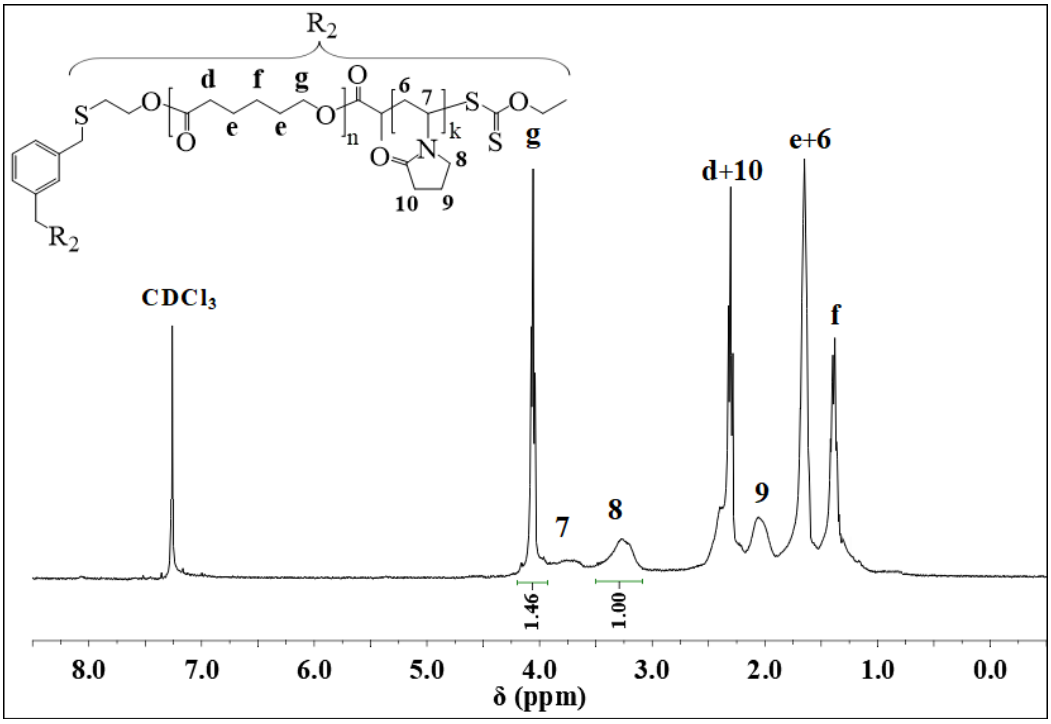

Figure S3. ^{1}H NMR spectrum of MXTP2 using MXTPCL-X1

M_n(NMR) of MXTP4 was calculated by comparing the peak integrals derived from the methyl protons' peak of PDMA (δ =3.22–2.72 ppm, peak "**23**") and the methylene protons' peak of PCL (δ = 4.03 ppm, peak "**g**") in Figure S4 according to (3) and (4)

$$M_n \text{ (NMR)}= DP_{PDMA}\, M_{monomer} + M_{n,MXTPCL-X2} \quad (3)$$

$$DP_{PDMA} = \frac{I_{23}}{12H}\, x \frac{4H}{I_g}\, x\ DP_{PCL} \quad (4)$$

Here, DP_{PDMA} and DP_{PCL} are degrees of polymerization for PDMA and PCL segments, respectively. $M_{monomer}$ is also the molecular weight of the DMA. M_n(NMR) was calculated 32290 g/mol.

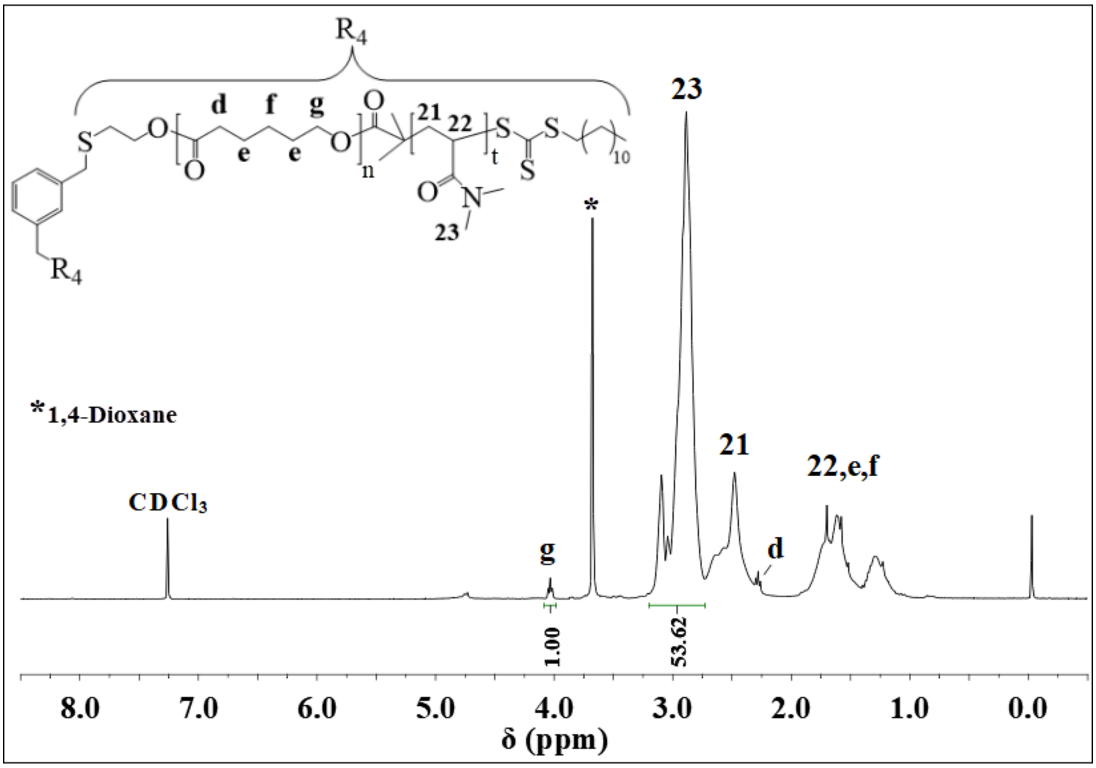

Figure S4. ^{1}H NMR spectrum of MXTP4 using MXTPCL-X2
